# Transcranial direct current stimulation enhances theory of mind in Parkinson’s disease patients with mild cognitive impairment: a randomized, double-blind, sham-controlled study

**DOI:** 10.1186/s40035-018-0141-9

**Published:** 2019-01-07

**Authors:** Mauro Adenzato, Rosa Manenti, Ivan Enrici, Elena Gobbi, Michela Brambilla, Antonella Alberici, Maria Sofia Cotelli, Alessandro Padovani, Barbara Borroni, Maria Cotelli

**Affiliations:** 1 Department of Psychology, University of Turin, Turin, Italy; 2 Neuroscience Institute of Turin, Turin, Italy; 3 Neuropsychology Unit, IRCCS Istituto Centro San Giovanni di Dio – Fatebenefratelli, Brescia, Italy; 4 Department of Philosophy and Educational Sciences, University of Turin, via Gaudenzio Ferrari 9, 10124 Turin, Italy; 5 Centre for Neurodegenerative Disorders, Neurology Unit, Department of Clinical and Experimental Sciences, University of Brescia, Brescia, Italy

**Keywords:** Medial frontal cortex (MFC), Mild cognitive impairment (MCI), Parkinson’s disease (PD), Theory of mind (ToM), Transcranial direct current stimulation (tDCS)

## Abstract

**Background:**

Parkinson’s Disease (PD) with mild cognitive impairment (MCI) (PD-MCI) represents one of the most dreaded complications for patients with PD and is associated with a higher risk of developing dementia. Although transcranial direct current stimulation (tDCS) has been demonstrated to improve motor and non-motor symptoms in PD, to date, no study has investigated the effects of tDCS on Theory of Mind (ToM), i.e., the ability to understand and predict other people’s behaviours, in PD-MCI.

**Methods:**

In this randomized, double-blind, sham-controlled study, we applied active tDCS over the medial frontal cortex (MFC) to modulate ToM performance in twenty patients with PD-MCI. Twenty matched healthy controls (HC) were also enrolled and were asked to perform the ToM task without receiving tDCS.

**Results:**

In the patients with PD-MCI, i) ToM performance was worse than that in the HC, ii) ToM abilities were poorer in those with fronto-executive difficulties, and iii) tDCS over the MFC led to significant shortening of latency for ToM tasks.

**Conclusions:**

We show for the first time that active tDCS over the MFC enhances ToM in patients with PD-MCI, and suggest that non-invasive brain stimulation could be used to ameliorate ToM deficits observed in these patients.

## Background

Parkinson’s Disease (PD) is the second most common neurodegenerative disease [1]. Its cardinal clinical manifestations are motors dysfunctions, including resting tremor, muscle rigidity, akinesia, and postural instability [2–6]. However, PD is also associated with non-motor deficits, including cognitive and emotional impairments, autonomic dysfunction, sleep disorders, neuropsychiatric disorders, and sensory impairment [3, 4, 7–16]. Recent studies have suggested that these symptoms are the major determinants of quality of life in patients with PD [13, 17].

In recent years, there has been great interest in the early phases of PD. In particular, PD-mild cognitive impairment (PD-MCI) represents a transitional state between normal aging and dementia in patients with PD. This condition is associated with a higher risk of developing PD with dementia and represents one of the most dreaded complications of the disease for patients and caregivers [18]. Interestingly, PD-MCI prevalence seems to range from 25 to 30% among non-demented patients with PD [19]. PD-MCI is a heterogeneous clinical condition and is generally characterized by impairments in cognitive domains such as memory, visuospatial function, and frontal/executive functions, with preserved activities of daily living [3, 4, 10, 19–23].

Research on the social cognitive profile of PD has revealed disturbances in Theory of Mind (ToM) ability, i.e., the ability to attribute mental states to others and to predict, describe, and explain behaviour on the basis of such mental states [24–29]. Imaging studies have suggested that ToM ability relies on a distributed neural network including the right and left temporo-parietal junctions, the precuneus, and the medial frontal cortex (MFC) [30, 31]. Several studies have suggested a pivotal role for the MFC in ToM (for a review, see [32]).

ToM has attracted considerable interest in neurodegenerative diseases in recent years [33–39]. In particular, ToM difficulties recorded in patients with PD in the early stages of the disease principally involve the cognitive component of ToM (i.e., inferences about others’ beliefs and intentions). The spatio-temporal progression of dopamine depletion in PD supports the hypothesis that the affective component (i.e., inferences about others’ emotions and feelings) may only be impaired in the advanced stages of the disease when depletion of dopamine in the striatum and the consequent hypostimulation of the MFC also involve more medial portions of this brain region (the dorsolateral portion of MFC is affected in early PD stages). Interestingly, it has been proposed that difficulties in the cognitive component of ToM, which are detected in ToM tasks in patients with PD, could be partially explained by the executive function impairment characterizing the disease [38].

In this study, we applied anodal transcranial direct current stimulation (tDCS) over the MFC (Fpz site, with the cathode between Oz and Inion) to modulate ToM performance in patients with PD-MCI. tDCS is a non-invasive stimulation technique wherein weak polarizing electrical current externally applied through a pair of electrodes attached to the head for a few minutes can generate a change in neuronal excitability and consequently modulate cognitive task performance. Depending on the polarity of the current flow, brain excitability can be increased (anode) or decreased (cathode) [40–42].

Several studies have reported short-term modulation of cognitive performance in typical populations and in patients using tDCS [43–45]. In addition, a single tDCS session has been demonstrated to have an effect on social cognitive abilities in healthy subjects [46–53]. Although anodal tDCS has already been demonstrated to improve motor and non-motor symptoms in PD [54–60], to the best of our knowledge, no study has yet investigated the effects of tDCS on ToM abilities in patients with PD-MCI.

To evaluate the impact of active tDCS on ToM performance in patients with PD-MCI, we used a video-tape version of a cognitive ToM task to investigate the ability to represent other individuals’ private and communicative intentions based on the observation of daily actions [61–64]. We investigated whether the application of active tDCS over the MFC (Fpz site, with the cathode between Oz and Inion) would facilitate ToM performance in this study with a double-blind, sham-controlled experimental design. Based on the PD cognitive profile described, we hypothesized that, in patients with PD-MCI, ToM task performance would i) be worse than it is in healthy controls, ii) correlate with executive functioning, and iii) be enhanced by active tDCS over the MFC when compared to sham.

## Materials and methods

### Participants and control group

Twenty patients fulfilling the UK Parkinson’s Disease Society Brain Bank clinical diagnostic criteria for PD [65, 66] were consecutively recruited at IRCCS Istituto Centro San Giovanni di Dio Fatebenefratelli in Brescia, Italy. Patients with PD-MCI were classified using the Parkinson’s Disease Cognitive Rating Scale (PD-CRS) (PD-MCI score range = 65–81 [67]) and using comprehensive neuropsychological testing to assess different cognitive domains *(*see below) [3].

The exclusion criteria included a) other neurological and psychiatric disorders; b) history of traumatic brain injury; c) clinically known hearing or vision impairment or a past history of alcohol abuse; d) diagnosis of PD-dementia [67] or Mini-Mental Parkinson (MMP) score < 25 [68, 69]; and e) any contraindication for tDCS [70]. All patients had been on stable pharmacological therapy for at least 6 months prior to entering the study.

Twenty matched healthy controls (HC) were also enrolled in the study for behavioural assessment of their ToM abilities (11 women and 9 men, mean age = 69.4 ± 5.8 years, mean education = 11.6 ± 4.4 years). The inclusion criteria for the HC were as follows: no history of mental illness or cognitive decline, no motor or cognitive complaints, normal objective cognitive performance in all of the administered neuropsychological tests, normal scores in functional assessment, and absence of mood and anxiety disorders. The HC were asked to perform the ToM task without tDCS in order to allow us to compare ToM abilities in patients with PD-MCI to those in HC.

All participants were made fully aware of the aims of the research, and written informed consent was obtained from all subjects. The study was approved by the local ethics committee (IRCCS Istituto Centro San Giovanni di Dio Fatebenefratelli, Brescia, Italy) and was conducted in accordance with the Declaration of Helsinki.

### Procedure

#### Clinical and neuropsychological assessment

All patients underwent extensive clinical and neuropsychological evaluations carried out over two sessions.

A comprehensive neuropsychological assessment based on the previously published recommendations is used [3, 69]. The cognitive tests battery included test for assessing global cognitive abilities (PD-CRS [71–73]) and tests for memory, language, and attentional and executive functions. We used MMP as a brief screening test for global cognition [66, 68, 74, 75]. In addition, PD-CRS is applicable to all stages of PD both for routine clinical practice and for data collection in clinical trials and it is rated as “recommended” by the International Parkinson and Movement Disorder Society [76–78].

The neuropsychological test battery included measures used to assess verbal fluency (phonemic and semantic), object and action naming (International Picture Naming Project, [79]), attention and executive functions (Trail Making Test, Test of Attentional Performance [TEA] [80], Stroop Test, Frontal Assessment Battery [FAB] [81]), and memory (Rey Auditory Verbal Learning Test, immediate and delayed recall, and Digit Span Forward and Backward). All of the tests were administered and scored according to standard procedures [82]. Results of the neuropsychological assessment of the patients with PD-MCI are presented in Table 1.

**Table 1:** Neuropsychological assessment of patients with Parkinson’s Disease-mild cognitive impairment (*n* = 20)

	Mean (SD)	Cut-off
Screening for dementia		
Mini Mental Parkinson	28.0 (2.7)	≥22.85
Global cognitive abilities		
Parkinson’s Disease-Cognitive Rating Scale (PD-CRS)		
PD-CRS Total Score (max: 134)	**78.9 (15.6)**	≥82
PD-CRS Cortical Score (max: 30)	26.1 (2.4)	–
PD-CRS Frontal Subcortical Score (max: 104)	52.8 (14.0)	–
Memory		
Rey Auditory Verbal Learning Test, immediate recall	35.8 (11.1)	> 28.52
Rey Auditory Verbal Learning Test, delayed recall	7.0 (3.5)	> 4.68
Digit Span (Forward)	5.5 (0.9)	> 4.25
Digit Span (Backward)	4.1 (0.9)	> 2.64
Language		
Verbal Fluency, phonemic	30.6 (9.9)	> 16
Verbal Fluency, semantic	37.2 (9.0)	> 24
Objects Picture Naming task (International Picture Naming Project (IPNP), %)	69.1 (11.9)	–
Actions Picture Naming task (IPNP, %)	87.3 (14.2)	–
Attentional and Executive Functions		
Frontal Assessment Battery	15.3 (2.5)	> 13.4
Stroop test (interference effect on time, seconds)	32.4 (14.1)	< 36.92
Stroop test (interference effect on error number)	2.4 (3.3)	< 4.24
Trail Making Test, part A (seconds)	51.8 (26.5)	< 94
Trail Making Test, part B (seconds)	184.7 (125.7)	< 283
Test of Attentional Performance		
Go/NoGo (time, ms)	528.9 (88.7)	–
Go/NoGo (accuracy)	28.2 (2.5)	–
Working Memory (time, milliseconds)	803.6 (192.5)	–
Working Memory (accuracy)	11.7 (1.1)	–
Response Flexibility (time, milliseconds)	1390.4 (512.2)	–
Response Flexibility (accuracy)	78.0 (17.8)	–

Raw scores are reported. Standard deviation (SD) is presented in parentheses

Cut-off scores according to Italian normative data are reported

Bold data indicate scores below the cut-off value

Depressive symptoms were assessed using the Beck Depression Inventory-II (BDI-II; [83]). Clinical evaluation included the Parkinson’s Disease Quality of Life Questionnaire-39 [84], the Barratt Impulsivity Scale [85], the Apathy Evaluation Scale [86], and the Rapid Eye Movement Sleep Behavior Disorders Screening Questionnaire (RBDSQ; [83–85, 87]). The Unified Parkinson’s Disease Rating Scale (UPDRS-III; [88]), the Italian version of the 20-Item Toronto Alexithymia Scale (TAS 20; [89]), the Interpersonal Reactivity Index [90], and the Hoehn & Yahr Scale [91] were also administered. The demographic and clinical characteristics of the patients with PD-MCI are presented in Table 2.

**Table 2:** Demographic and clinical characteristics of patients with Parkinson’s Disease-mild cognitive impairment (*n* = 20)

	Mean (SD)
Age (years)	65.6 (8.4)
Education (years)	10.3 (4.6)
Sex (male/female)	10/10
Age of onset (years)	58.9 (7.5)
Levodopa-equivalent daily dose (mg)	555.7 (323.1)
Cumulative Illness Rating Scale – severity	1.6 (0.3)
Cumulative Illness Rating Scale – comorbidity	2.9 (1.8)
Unified Parkinson’s Disease Rating Scale III	24.1 (9.5)
Hoehn & Yahr	1.8 (0.7)
Toronto Alexithymia Scale-20	50.7 (14.8)
Interpersonal Reactivity Index	84.4 (13.0)
REM Sleep Behavior Disorder Screening Questionnaire	3.6 (2.4)
Parkinson’s Disease Quality of Life Questionnaire	23.2 (13.0)
Beck Depression Inventory II	11.7 (6.0)
Apathy Evaluation Scale	12.1 (10.2)
Barratt Impulsivity Scale-11	60.5 (8.1)

Standard deviation (SD) is presented in parentheses

The present work was a randomized, double-blind, sham-controlled study. Each patient received both active and sham tDCS over the MFC (Fpz site, with the cathode between Oz and Inion) in two different sessions separated by at least 2 days and in randomized order. All patients and the experimenter were blind to the type of tDCS applied. During each session, a video version of a cognitive ToM task was administered. According to the literature, tDCS excitability changes induced by one session of tDCS applied for a few minutes are expected to last for up to 1 h [92–94]. Therefore, the performance of the patients was expected to return to its initial level between the two sessions of stimulation.

#### Theory of mind tasks

All of the participants performed two ToM tasks: the Reading the Mind in the Eyes (RME) task, which was used to assess individual ToM abilities, and the Attribution of Intentions (AI) task, which was used to test the effects of tDCS on cognitive ToM ability.

The RME is an advanced ToM task evaluating the subject’s ability to represent others’ mental states by observing only their eyes [95]. The participants were required to choose which word, among four options, better described what the character in the photograph was thinking or feeling. The total number of correct choices represented the RME score. Participants were administered the RME before the tDCS brain stimulation.

**Fig. 1:**
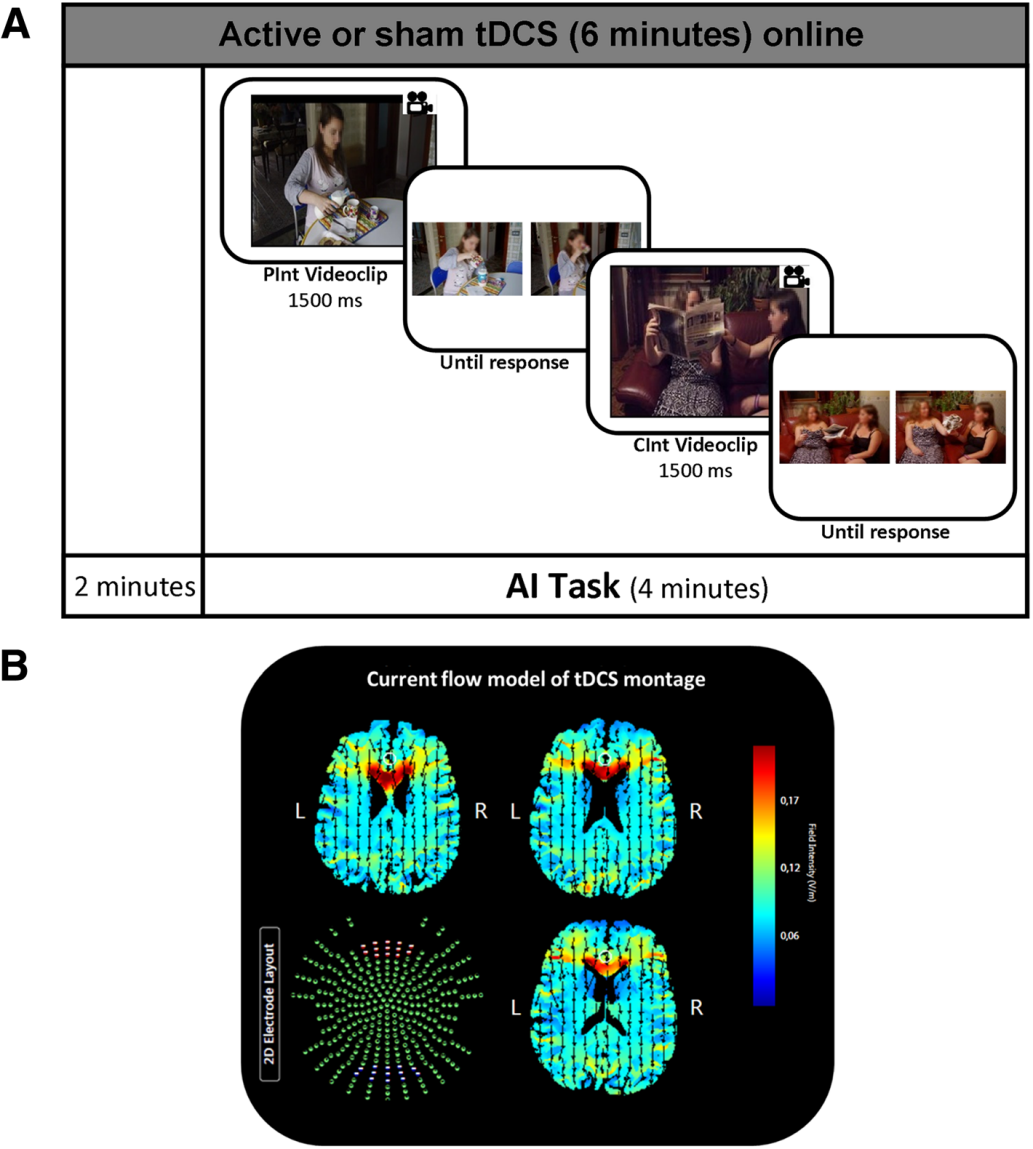
**a** Experimental design. Active or sham tDCS was started 2 min before the beginning of the experimental ToM block and continued throughout the AI task. In the AI task, a short video was played, and the participant was asked to choose the picture representing a logical story ending by pushing one of the two buttons on the button box. One example for each stimulus condition (CInt and PInt) is displayed. **b** Current flow model for tDCS. The anode was placed over the medial frontal cortex and the cathode was placed between Inion and Oz. The device utilized two 7- × 5-cm sponge pads represented in the transverse view on the Male 1 model in Soterix HD Targets software (Soterix Medical). Arrows represent the direction of current flow

The AI task is a video version of a cognitive ToM task previously used in young healthy individuals [46]. Participants were asked to demonstrate their comprehension of the displayed stories (short videos lasting 1500 milliseconds) by choosing the appropriate story ending (out of two concluding pictures displayed until the response). The two possible story endings were shown simultaneously until the participant responded by pressing the corresponding button on the button box as quickly as possible. The correct picture represented a probable conclusion, whereas the incorrect picture represented an improbable ending (see Fig. 1).

The two experimental conditions were a) the Private Intention condition (PInt), in which participants were required to recognize another person’s intention while watching his/her isolated actions, e.g., hanging a picture on the wall; and b) the Communicative Intention condition (CInt), in which participants were required to recognize another person’s communicative intention during a social interaction, e.g., asking another person to obtain a glass of water for them.

We displayed 34 video stories for each condition, for a total of 68 stories using Presentation software (Version 16.3, www.neurobs.com) running on a personal computer with a 15-in. screen. Participants were seated in a quiet room in front of a computer monitor placed 60 cm away from them. The visual location (right and left side of the screen) of the correct answer was randomized. Accuracy was recorded as the number of correct trials. The reaction time (RT) for each correct response was recorded in milliseconds from the onset of the presentation of the two concluding pictures until detection of the response. The items were divided into two blocks (17 PInt and 17 CInt stimuli each) corresponding to the two types of stimulation (active and sham stimulation). Two additional stimuli for each condition were selected for use in a training session. The stimulation conditions and the order of the presentation of the two blocks were randomized across participants. The tests were administered on two consecutive days at the same time of the day to minimize the likelihood of interference from confounding factors.

#### tDCS procedure

Active tDCS was applied using a battery-driven constant-current stimulator (BrainStim, EMS; Bologna, Italy) through a pair of saline-soaked sponge electrodes (7 cm × 5 cm). The target area for tDCS was the MFC (Montreal National Institute coordinates: 0, 60, 18), as it has been recognized as a pivotal region in intention processing [62–64, 96, 97]. In order to apply tDCS to the MFC, we placed the anode over Fpz and the cathode between Inion and Oz (Fig. 1) according to the 10–20 electroencephalography international system [98]. During active tDCS, a constant current of 1.5 mA was applied for 6 min (with a ramping period of 10 s at the beginning of the stimulation), starting 2 min before the beginning of the AI task and covering the entire duration of the task. The current density (0.043 mA/cm^2^) was maintained below the safety limits [70]. In the sham stimulation condition, the tDCS procedure was the same, but the current was turned off 10 s after the beginning of the stimulation (not including the durations of the fade-in and fade-out periods = 10 s) and turned on for the last 10 s of the stimulation period. Therefore, the participants experienced an itching sensation below the electrodes at the beginning and end of the stimulation, making this condition indistinguishable from the experimental stimulation.

The study was a randomized double-blind experiment: the participants and the experimenter were not aware of the stimulation that was delivered. The two AI stimuli sessions corresponded to the two tDCS conditions: active and sham tDCS. The stimulation conditions were randomized across participants and executed on two different days at the same time of day to minimize the likelihood of interference from confounding factors. Half of the male and female participants received active stimulation on day 1 and sham stimulation on day 2, while the other half of the participants received sham stimulation on day 1 and active stimulation day 2. Active or sham tDCS were delivered after a numeric code was input into the device. This step allowed for blinding of the operator before and during the tDCS administration.

In order to detect differences in the perception of sensations, to blind the participants to the type of stimulation they were receiving, and to register potential side effects of tDCS, at the end of the stimulation session we asked the participants to answer a questionnaire regarding the perceptual sensations they experienced during the active and sham tDCS experiments.

#### Statistical analyses

Statistical analyses were performed using Statistica software (version 10; www.statsoft.com/Products/STATISTICA-Features). Since the data were normally distributed (RTs, Kolmogorov-Smirnov Test: d = 0.19, *p* = 0.21; and accuracy, Kolmogorov-Smirnov Test: d = 0.16, *p* > 0.25), AI task performance (accuracy and RT) was analysed using parametric analyses.

First, AI task performance (accuracy and RT) in the PD-MCI group under the sham tDCS condition was compared to that in an age-, sex-, and education-matched HC group using analysis of variance (ANOVA) with the type of stimulus (PInt or CInt) as the within-participant factors and group (PD or HC) as the between-participants factors. We compared the RME scores of the PD-MCI and HC groups using a one-way ANOVA.

Subsequently, PD-MCI AI task performance (accuracy and RT) was analysed using repeated-measures ANOVA, with the type of stimulation (active or sham) and the type of stimulus (PInt or CInt) as within-participant factors, and sex as the between-participants factor. Sensation scores were compared between active tDCS and sham tDCS using a Wilcoxon matched-pair test. Statistical significance was set at *p* < 0.05.

Finally, a regression analysis was performed for correlations between ToM performance (AI task mean performance in the sham tDCS condition and the RME score) and neuropsychological assessment measures in order to investigate the relationship between ToM and cognitive abilities. We also performed regression analysis between active tDCS-induced changes in the AI task (RTs in the sham tDCS condition subtracted by those in the active tDCS condition) and neuropsychological assessment measures to determine whether greater difficulties in some cognitive abilities influenced the effects of tDCS on ToM. Statistical significance for regression analysis was set at *p* < 0.01 (Bonferroni corrected for the number of comparisons, *p* = 0.05/5 = 0.01) for clinical scales, at *p* < 0.013 (Bonferroni corrected for the number of comparisons, *p* = 0.05/4 = 0.013) for assessments of memory and language and at *p* < 0.005 (Bonferroni corrected for the number of comparisons, *p* = 0.05/11 = 0.005) for attentional and executive functions evaluations.

Statistical power and effect size (Cohen’s d) analyses were performed using GPower 3.1 [99]. The datasets used and/or analysed during the current study are available from the corresponding author on reasonable request.

## Results

### Behavioural performance on ToM tasks in patients with PD-MCI and HC

#### RME task

**Fig. 2:**
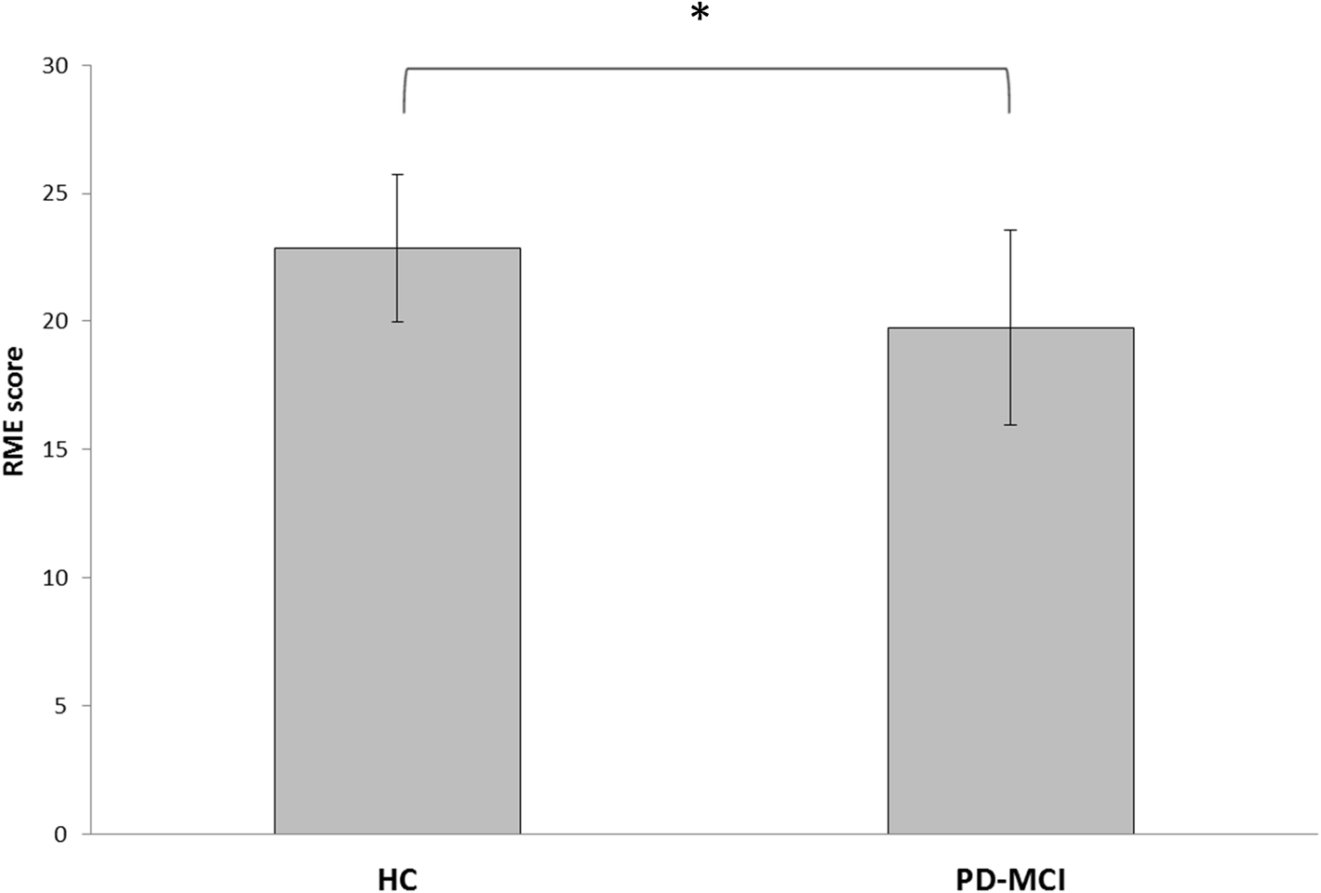
Score obtained in the Reading the Mind in the Eyes task in patients with PD-MCI and HC plotted separately. The patients with PD-MCI had significantly worse accuracy than the HC did. Asterisks indicate significant effects (*p* < 0.05)

Analysis of the RME scores revealed a significant effect of group (F_(1,38)_ = 8.03, *p* = 0.007, η^2^ = 0.17, 1-β = 0.99). This result indicates that the PD-MCI group performed worse than the HC group in this task (HC: 22.9 ± 3; PD-MCI: 19.7 ± 4). See Fig. 2 for details.

#### AI task

Accuracy analysis indicated significant effects of group (F_(1,38)_ = 8.53, *p* = 0.006, η^2^ = 0.18, 1-β = 0.99) and the type of stimulus (F_(1,38)_ = 4.84, *p* = 0.033, η^2^ = 0.11, 1-β = 0.99). These results indicate that accuracy in the CInt condition was significantly worse than that in the PInt condition in both groups, and that the PD-MCI group had less accuracy in this task than the HC group did (HC, CInt: 87.1% ± 12, PInt: 94.4% ± 6; PD-MCI, CInt: 79.1% ± 16%, PInt: 80.3% ± 16%).

**Fig. 3:**
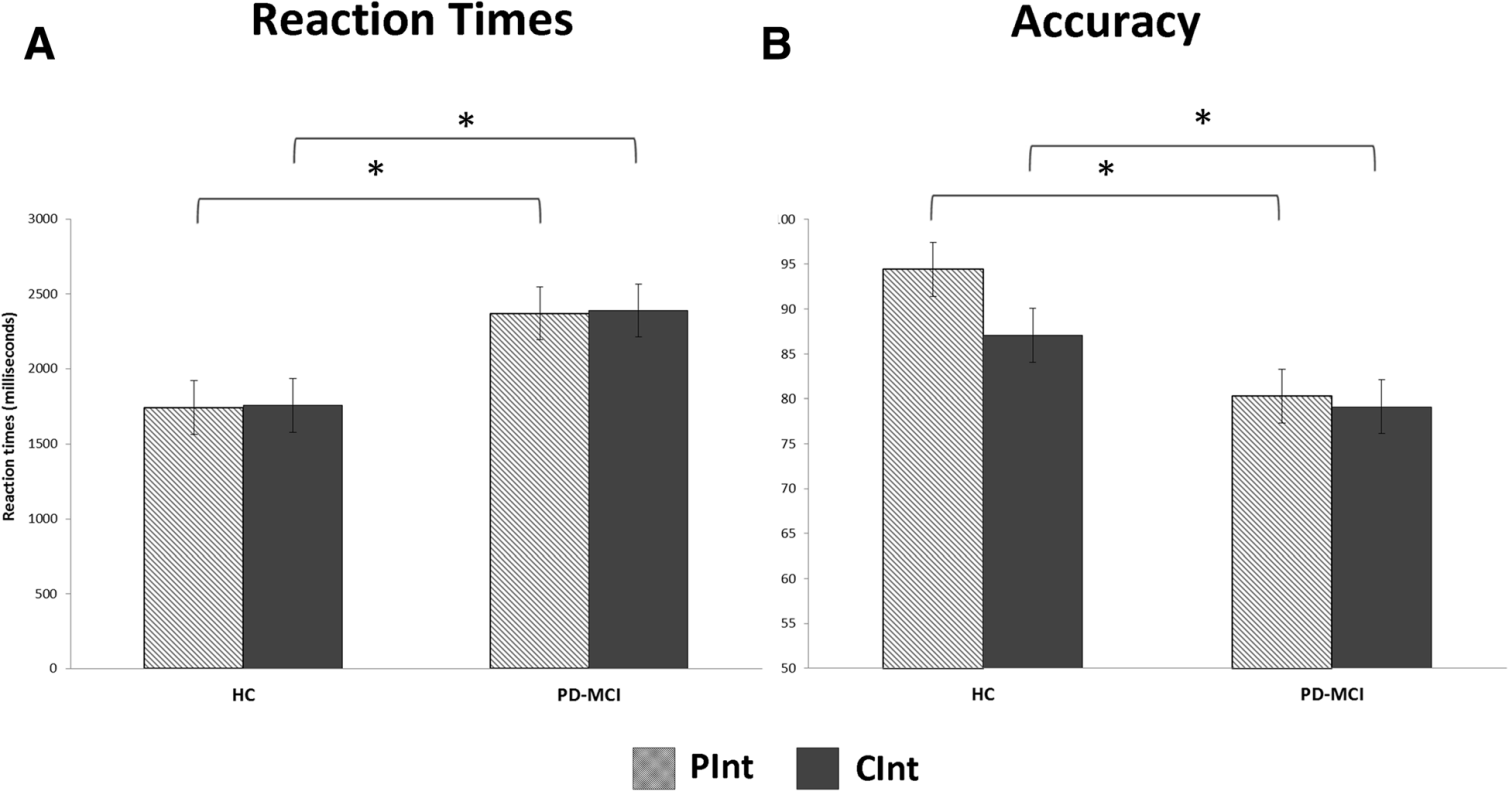
Reaction times **a** and accuracy **b** in the AI task (CInt and PInt conditions) in patients with PD-MCI and HC plotted separately. The patients with PD-MCI had significantly worse accuracy and RTs in the CInt and PInt conditions than the HC did. Asterisks indicate significant effects (*p* < 0.05)

RT analysis revealed a significant effect of group (F_(1,38)_ = 6.31, *p* = 0.016, η^2^ = 0.14, 1-β = 0.99). This result indicates that the PD-MCI group was slower than the HC group in both CInt and PInt conditions (HC, CInt: 1757.0 ± 387 ms, PInt: 1742.2 ± 321 ms; PD-MCI, CInt: 2391.1 ± 1042 ms, PInt: 2370.5 ± 1084 ms). No other effect reached statistical significance. See Fig. 3 for details.

### Effects of tDCS on the AI task in patients with PD-MCI

Accuracy analysis revealed no significant effects of the type of stimulation (F_(1,18)_ = 1.31, *p* = 0.27, η^2^ = 0.06, 1-β = 0.18), the type of stimulus (F_(1,18)_ = 1.37, *p* = 0.26, η^2^ = 0.07, 1-β = 0.65), or sex (F_(1,18)_ = 3.87, *p* = 0.07, η^2^ = 0.17, 1-β = 0.65), or interactions between factors (CInt condition: 79% ± 16% [sham tDCS], 75% ± 18% [active tDCS]; PInt condition: 80% ± 15% [sham tDCS], 78% ± 18% [active tDCS]).

**Fig. 4:**
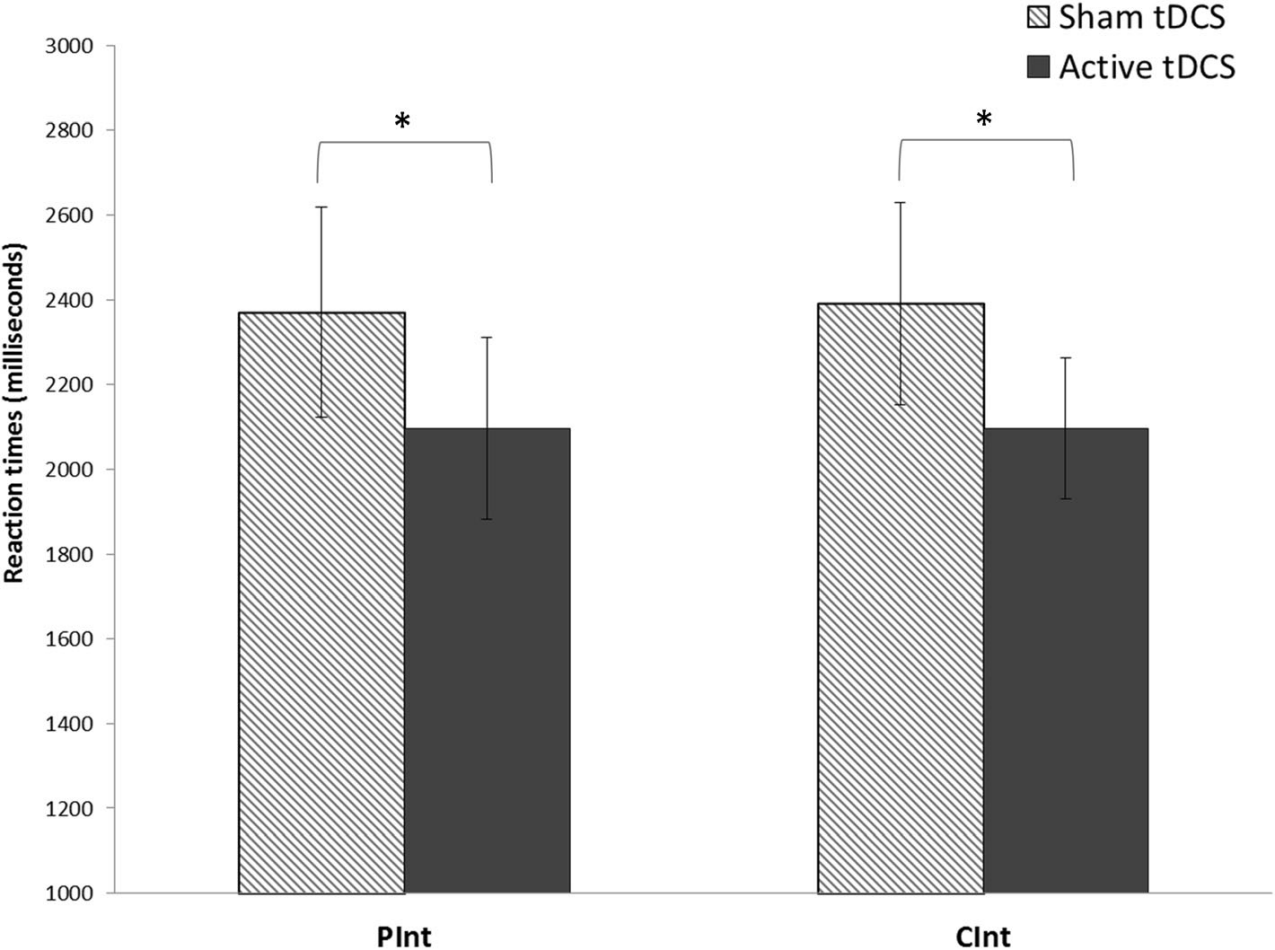
Effects of tDCS on reaction times in the AI task in patients with PD-MCI under the active tDCS and sham tDCS conditions (CInt and PInt conditions plotted separately). The reaction times of patients with PD-MCI in the AI task decreased after active tDCS over the MFC (Fpz site, with the cathode between Oz and Inion) when compared to sham stimulation in both the CInt and Pint conditions. Asterisks indicate significant effects (*p* < 0.05)

RT analysis revealed a significant effect of the type of stimulation (active vs. sham) (F_(1,18)_ = 6.21, *p* = 0.022, η^2^ = 0.26, 1-β = 0.99). No other effect reached statistical significance. These results indicate active tDCS induced shorter RTs than sham tDCS in both CInt and PInt conditions (Active tDCS, CInt: 2096.1 ± 727 ms, PInt: 2096.0 ± 939 ms; Sham tDCS, CInt: 2391.1 ± 1042 ms, PInt: 2370.5 ± 1084 ms) (Fig. 4).

### Sensations questionnaire

Responses to the sensations questionnaire completed by patients with PD-MCI at the end of each stimulation session revealed that all of the patients tolerated the stimulation well. A Wilcoxon matched pairs test revealed that perceptual sensations reported after the active and sham stimulation sessions were not significantly different (*T* = 13.5, z = 1.73; *p* = 0.08). There was thus no reason to reject the blinded nature of this study.

### Correlation analysis between ToM performance and apathy, alexithymia, and neuropsychological assessment in patients with PD-MCI

We used ToM performance (AI task in sham tDCS condition and RME score) as the predictor variable and apathy, alexithymia, or neuropsychological assessment as the criterion variable. No significant correlations were found with RME score as the predictor variable.

**Fig. 5:**
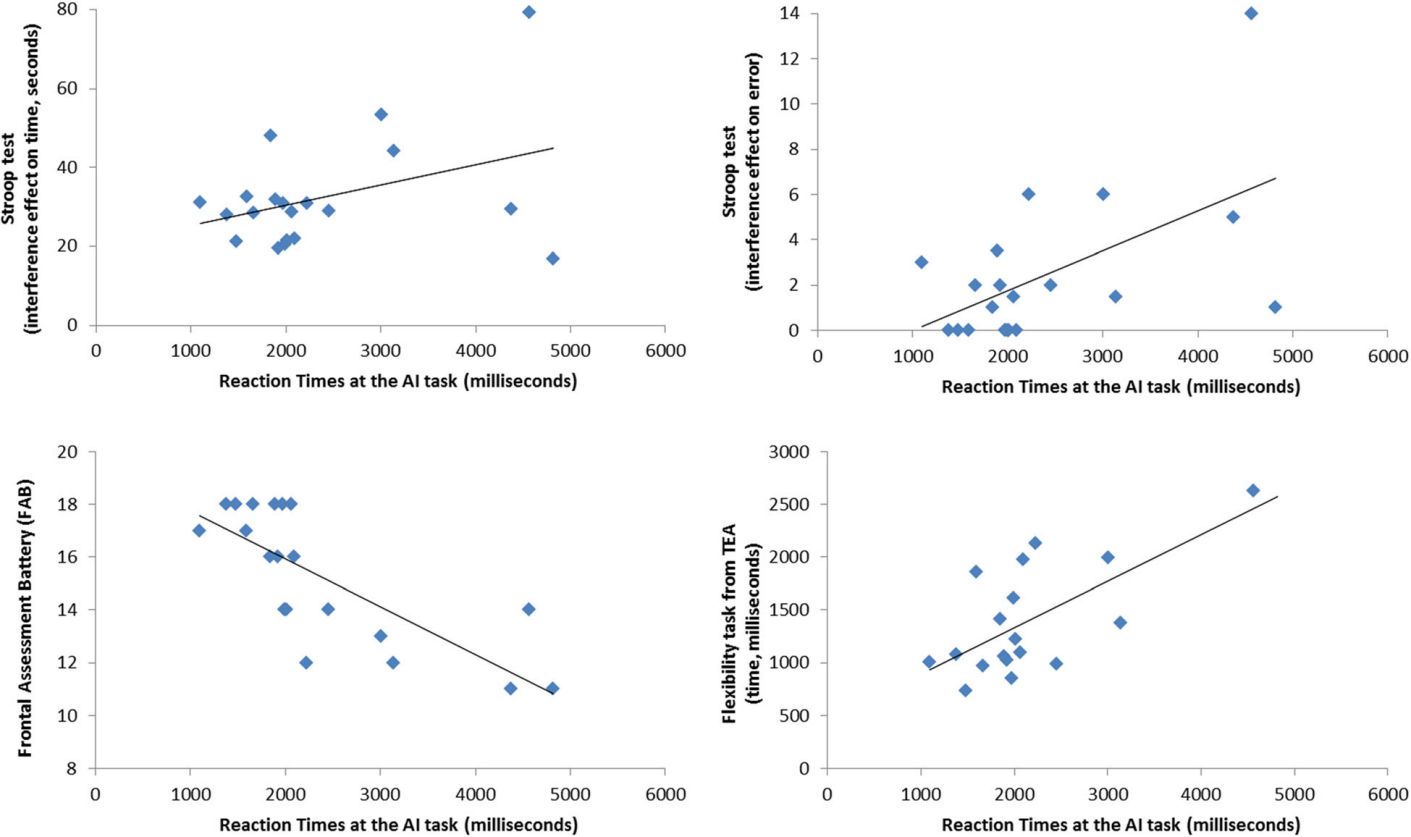
Significant correlations between reaction time of the Attribution of Intentions task in sham transcranial direct current stimulation condition and attentional-executive abilities in patients with PD-MCI

AI task RT predicted performance on the Stroop task (Time: *r* = 0.80, *p* < 0.0001; Errors: *r* = 0.79, *p* < 0.0001), performance on the FAB (*r* = − 0.67, *p* = 0.003) and time score of the Response Flexibility task of TEA battery (*r* = 0.70, *p* = 0.002), with poorer frontal-executive abilities associated with longer RTs in the AI task (see Fig. 5). No significant correlations were found between AI task accuracy and apathy, alexithymia, or other neuropsychological scores.

### Correlation analysis between active tDCS-induced changes in the AI task and apathy, alexithymia, and neuropsychological assessment in patients with PD-MCI

Apathy, alexithymia, or neuropsychological assessment was used as the predictor variable, and active tDCS-induced changes in the AI task were used as the criterion variable.

No significant correlations were found.

## Discussion

The aims of the present study were threefold. We hypothesized that, in patients with PD-MCI, ToM task performance would i) be worse than that in HC, ii) correlate with executive functioning, and iii) be enhanced by active tDCS over the MFC when compared to sham. Our results support the above hypotheses, as follows: i) In the communicative intentions and private intentions ToM tasks, the patients were slower and less accurate than the HC. ii) We found relationships between ToM and executive functioning. In particular, ToM abilities were poorer in patients with fronto-executive difficulties as assessed by the FAB and the Stroop task. iii) In patients with PD-MCI, a single session of active tDCS over the MFC led to significant shortening of latency in ToM tasks when compared to sham stimulation. We found no effects of tDCS on accuracy.

Until now, no study had investigated the effects of tDCS on ToM abilities in patients with PD-MCI, and only a few researchers have used tDCS in patients with PD. These researchers mainly focused on the treatment of motor [54, 57, 100–104] and cognitive symptoms [55, 58, 59, 105].

Our findings corroborate previous studies showing marked difficulties in the domain of ToM in patients with PD [24–29]. In particular, while neuropsychological studies have reported heterogeneous and controversial findings regarding affective ToM ability in patients with PD [106], there is consistent evidence for significant cognitive ToM impairments in these patients [26, 27, 107–109]. As recently shown by Bora, Walterfang, and Velakoulis [110], this may partially be explained by frontal-executive difficulties, which represent a crucial non-motor symptom of patients with PD-MCI. There is a significant relationship between ToM and executive functioning, even though the exact nature of this relationship between these two distinct domains remains at least partially unknown [111, 112]. At any rate, evidence from neuropsychological studies on ToM abilities suggests that executive functioning is necessary to perform cognitive ToM tasks [113–116]. Again, this observation is in line with our finding showing, in a sample of patients with PD-MCI, significant correlations between the RT to the AI task and both the FAB score and the interference effect on time and on error of the Stroop task.

Several lines of evidence suggest that the MFC might be critical for social cognition [46, 117, 118] and that increased MFC activity might result in enhanced social cognition [46]. The mechanisms underlying the effects of tDCS are not yet entirely understood, but might involve changes in the activities of some neurotransmitters [42, 119]. TDCS modifies the synaptic microenvironment by modifying synaptic strength (i.e., N-methyl-D-aspartate receptor functioning) or altering gamma-aminobutyric acid-mediated activity. It induces transient changes in the densities of protein channels localized to the cortical area below the electrodes, which would in turn interfere with brain excitability through the modulation of intracortical and corticospinal neuron activity [41, 42, 119]. In particular, the activities neurotransmitters, especially that of dopamine, seem to influence the plasticity changes induced by tDCS, as they depend on the relationship between current and the functioning of neurotransmitter receptors [119–121].

The finding that a single session of active tDCS over the MFC had a significant effect on ToM performance in patients with PD-MCI might have important clinical implications. In fact, evidence for enhancement of ToM performance by tDCS might lead to a potential intervention approach for this disease. However, we acknowledge that the present study has some limitations. First, it is important to underline that the enhancement we found was limited to RTs and that we observed no effect on accuracy. Another shortcoming is that we classified PD-MCI through a global scale (PD-CRS; [3]). A limitation in terms of the stimulation protocol is that we applied a single-session online tDCS design that focuses on short-term improvements induced by a single session of stimulation, typically delivered on-line during the task. The adoption of multiple sessions of tDCS could be used to investigate the long-term effects of stimulation, which are particularly interesting in neurodegenerative patients. Hence, further studies are needed in order to conclusively demonstrate the potential for the induction of long-term neuromodulatory effects using brain stimulation. In particular, the same protocol might be applied to PD-MCI patients to elucidate long-term improvements in ToM performance. Moreover, future studies including larger cohorts of patients at different stages of disease should be conducted before firm conclusion can be drawn. These interesting studies could better investigate the possibility to induce also effects on accuracy in addition to reaction times modulation. As known, tDCS is a non-invasive brain stimulation technique characterized by limited focality and several methodological factors, such as type of protocols design, target area, polarity and number of sessions, that can potentially influence the variability of behavioural and physiological outcomes [42, 122]. Accordingly, a further limit is represented by the lack of a control active stimulation site that should be considered in future studies to further confirm the specificity of the MFC for the present results on ToM tasks.

## Conclusion

Despite the limitations described, our findings show for the first time that active tDCS applied over the MFC could be useful for enhancing cognitive ToM ability in patients with PD-MCI. Our findings suggest that non-invasive brain stimulation could be used in an attempt to ameliorate the ToM deficits observed in these patients. Of course, further studies are needed to determine whether the effects found here are clinically meaningful. Nevertheless, the present study significantly contributes to the emergent evidence suggesting that tDCS may be used to treat PD [44, 123] and to improve social cognition [51, 124].

## Abbreviations

BDI-II: Beck Depression Inventory-II
FAB: Frontal assessment battery
HC: Healthy controls
MFC: Medial frontal cortex
MMP: Mini-Mental Parkinson
PD: Parkinson’s disease
PD-CRS: Parkinson’s disease cognitive rating scale
PD-MCI: Parkinson’s disease-mild cognitive impairment
RBDSQ: Rapid eye movement sleep behavior disorders screening questionnaire
TAS-20: Toronto Alexithymia Scale
tDCS: transcranial Direct Current Stimulation
TEA: Test of attentional performance
ToM: Theory of mind
UPDRS: Unified Parkinson’s Disease Rating Scale
